# Developing Odontoma in the Mandible of an Eight-Year-Old Boy

**DOI:** 10.7759/cureus.56230

**Published:** 2024-03-15

**Authors:** Sandeep Singh Sihmar, Shalini Rathi, Karthikeyan Ramalingam, Pratibha Ramani, Sathya Sethuraman

**Affiliations:** 1 Oral Pathology, Daswani Dental College, Kota, IND; 2 Oral Pathology, Darshan Foundation, Bhiwani, IND; 3 Oral Medicine and Radiology, Maharaja Ganga Singh Dental College and Research Centre, Sri Ganganagar, IND; 4 Oral Medicine and Radiology, Darshan Foundation, Bhiwani, IND; 5 Oral Pathology and Microbiology, Saveetha Dental College and Hospitals, Saveetha Institute of Medical and Technical Sciences, Saveetha University, Chennai, IND; 6 Dentistry, Saveetha Dental College and Hospitals, Saveetha Institute of Medical and Technical Sciences, Saveetha University, Chennai, IND

**Keywords:** developing odontoma, odontoma, non-calcifying odontoma, modified gallego, van gieson, hamartoma, mandibular molar region, radiolucency, impacted teeth, pediatric pathology

## Abstract

Benign mixed odontogenic tumors have been repeatedly classified and reclassified over the past few decades. Odontoma is considered a hamartoma due to its slow growth and non-aggressive nature. We present an interesting case of developing odontoma in an eight-year-old boy. His complaint was a slow-growing swelling in the lower right back tooth region. Clinical examination revealed a carious deciduous second molar. The orthopantomogram revealed a well-defined radiolucency around the unerupted mandibular first premolar and impacted mandibular second premolar. Histopathology revealed an odontogenic epithelial lining overlying myxofibrous stroma with inflammatory cells and calcified structures with few odontogenic rests. Special staining methods including Van Gieson and modified Gallego stains led to the final diagnosis of a developing odontoma.

## Introduction

As per the 2017 WHO classification, ameloblastic fibroma, primordial odontogenic tumor, compound composite odontoma, complex composite odontoma, and dentinogenic ghost cell tumor come under the category of benign epithelial and ectomesenchymal mixed odontogenic tumors. Varied degrees of inductive changes and dental hard tissue formation are noted in these tumors [1,2]. The true nature of these lesions is not deciphered yet. Ameloblastic fibroma is considered to be a true neoplasm, whereas odontoma is considered to be a hamartoma [3].

It was shown that ameloblastic fibroma resembles a developing tooth organ and could not be differentiated from a non-calcifying odontoma [1,3]. Chrcanovic and Gomez [4] have reported in their systematic review that the concept of progressive maturation of ameloblastic fibroma into an odontoma does not happen in all cases. There are situations where the diagnosis of odontoma becomes difficult. We report the case of an unusual odontoma that radiographically mimics an odontogenic cyst. The confirmatory diagnosis was made after histopathological analysis and using special stains.

## Case presentation

**Figure 1:**
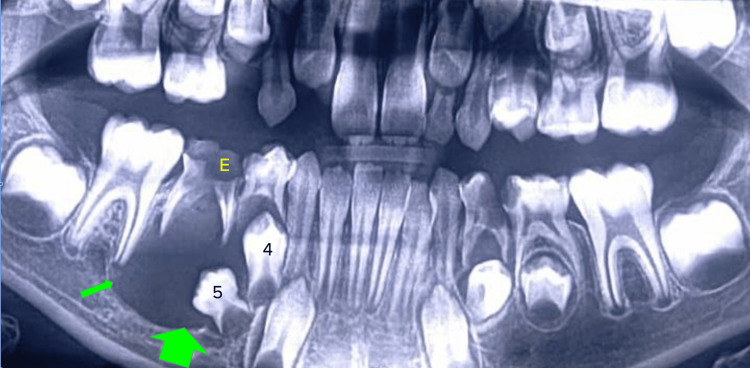
Orthopantomogram showing the radiolucency in the right mandibular premolar region Green arrows: radiolucency; E: carious deciduous second molar; 4: first premolar; 5: second premolar

An eight-year-old male patient reported to Darshan Dental Clinic, Bhiwani, India, with the chief complaint of swelling in the lower right back tooth region for three months. The swelling was initially small in size and gradually increased with time. Intraoral examination revealed dental caries in the right lower primary second molar tooth, 85. The orthopantomogram revealed a well-defined radiolucency around the crown of unerupted mandibular premolars. It was a well-defined, unilocular radiolucency in the apical region of 85 involving the coronal portion of unerupted 44 and completely involving impacted 45 until the inferior alveolar nerve level (Figure 1).

The provisional diagnosis of an odontogenic cyst was made. Aspiration was negative from the lesion. Hence, surgical enucleation of the lesion was performed under local anesthesia along with the removal of the associated mandibular first and second premolars. The excised specimen was sent to the Oral Pathology Department, Saveetha Dental College and Hospitals, for further processing.

**Figure 2:**
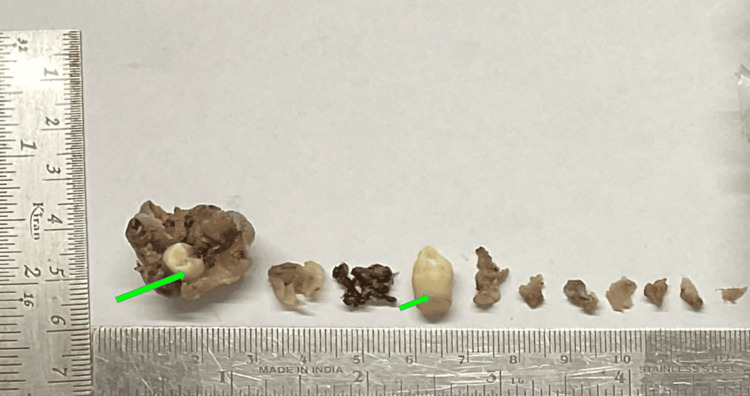
Grossing image showing the excised specimen with hard and soft tissues Green arrows: hard tissue sample and teeth removed during the surgery

Grossing of the received specimen was performed. The gross specimen shows multiple bits of hard and soft tissue specimens. The largest bit measured 3.1×2.0×1.6 cm (Figure 2).

**Figure 3:**
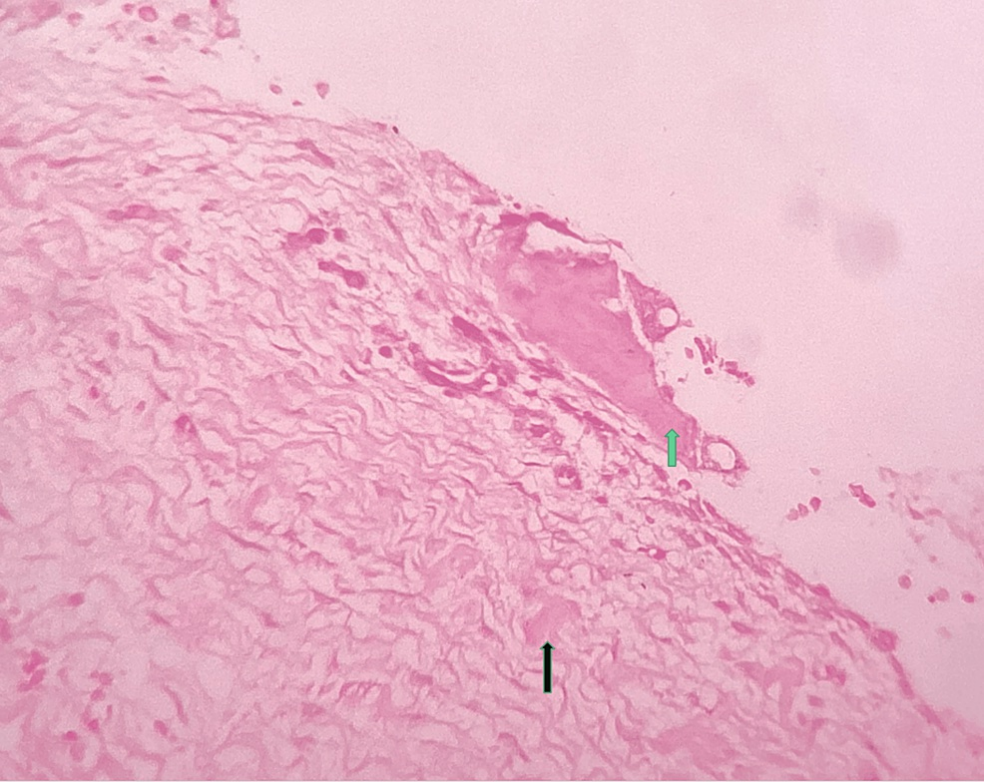
Photomicrograph showing fibro-myxoid stroma with eosinophilic calcifications (hematoxylin and eosin stain, 40×) Green arrow: eosinophilic calcification; black arrow: hyalinized areas within the stroma

The involved tooth in the cystic wall was kept for decalcification along with minimal attached soft tissue under 10% formic acid. The rest of the specimen was kept for routine tissue processing, sectioning, and staining. Histopathology showed odontogenic epithelial lining comprised of non-keratinized stratified squamous epithelium of two- to three-cell-layer thickness. The underlying connective tissue stroma was myxofibrous in nature. Foci of epithelial proliferation were noted in areas with intense inflammatory cell infiltration. Few odontogenic epithelial cell rests and eosinophilic calcifications were also evident (Figure 3).

**Figure 4:**
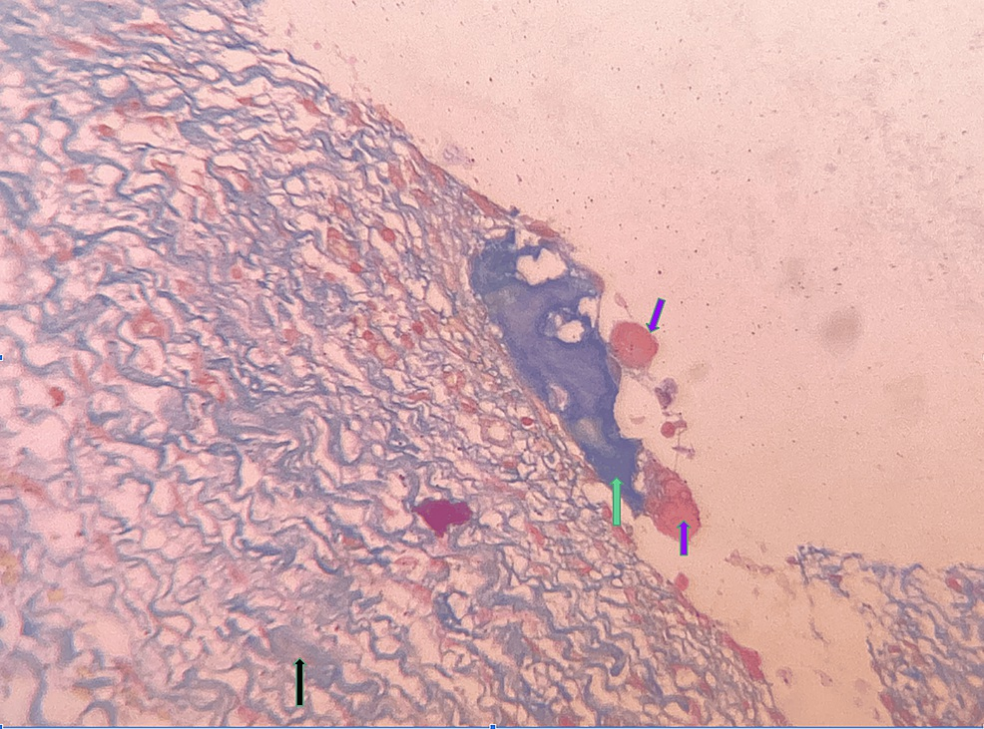
Photomicrograph showing pink cementum-like areas and blue-green dentin-like areas (modified Gallego stain, 40×) Black arrow: hyalinized areas in the stroma; green arrow: dentinoid areas; purple arrow: cementoid areas

The working diagnosis was given as an inflammatory odontogenic cyst. After staining with Van Gieson and modified Gallego, the final diagnosis was given as developing odontoma. Van Gieson stain revealed the epithelial component and muscles in yellow color while the collagen stained red. The modified Gallego stain stained the hard tissues in a blue color, and dentin-like areas showed a green color (Figure 4).

**Figure 5:**
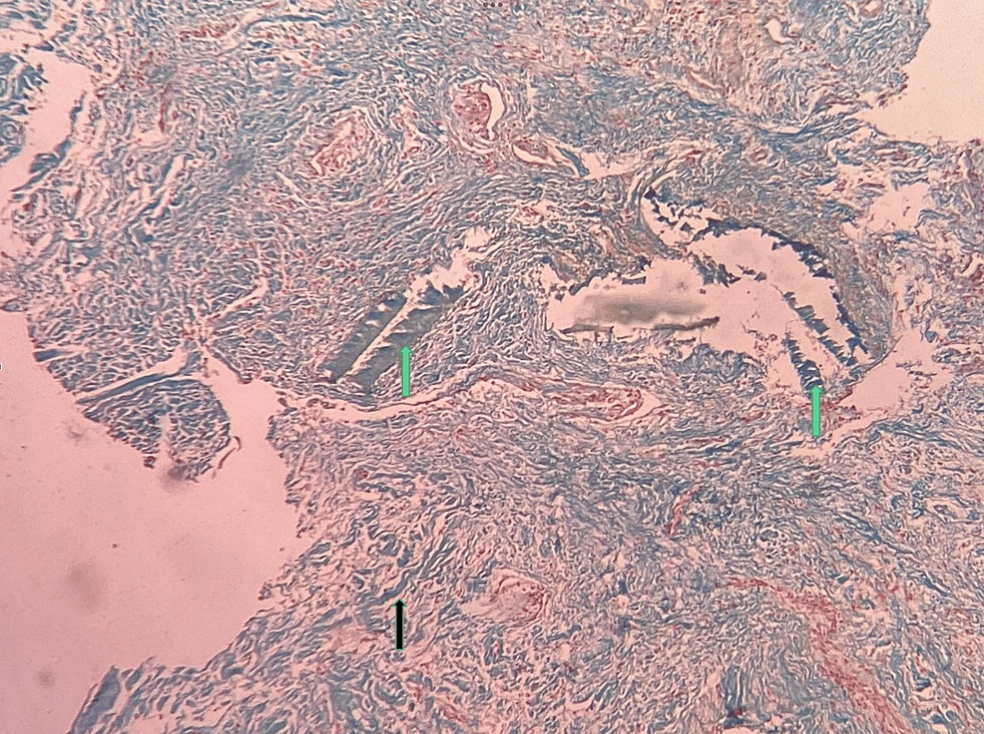
Photomicrograph showing mineralized areas within the sample (modified Gallego stain, 20×) Black arrow: hyalinized areas in the stroma; green arrow: dentinoid areas

Mineralized areas and hyalinized areas within the fibro-myxoid stroma showed a blue-green color suggestive of dentinoid material (Figure 5).

Correlating the clinical and radiological findings along with the histopathological features, the final diagnosis was given as a developing odontoma. There is no recurrence after 12 months of follow-up, and the patient is asymptomatic. 

## Discussion

Ameloblastic fibro-odontoma was redesignated as a developing odontoma in the 2017 WHO classification [5]. Bilodeau and Hunter [6] have recommended that clinicians should be familiar with various pediatric pathologies. Atarbashi-Moghadam et al. [7] reported that the mean age was 13.75 years, there was a male predominance, and asymptomatic swelling was the clinical presentation in their systematic review of ameloblastic fibro-odontoma. Prasad et al. [8] have presented a complex odontoma with ameloblastic follicles in a four-year-old child. Buchner et al. [9] reported mandibular predilection with posterior jaw involvement and a radiolucency around an unerupted tooth. Our patient was an eight-year-old boy who presented with similar clinical and radiological findings.

The dispute still exists whether ameloblastic fibroma, ameloblastic fibro-odontoma, and odontoma are separate neoplasms or hamartoma or do they belong to a single spectrum. Sanjai et al. [10] have suggested disease progression, while Chrcanovic and Gomez [4] denied this concept. Soluk-Tekkesin and Vered [5] have recommended further molecular and genetic analysis to understand the pathogenesis of this unique entity.

Benign mixed odontogenic tumors show epithelial and ectomesenchymal components. It is seen along with variable inductive changes and hard tissue formation. Satheesan et al. [11], Krishnakumar et al. [12], Afroze et al. [13], and Mudhiraj et al. [14] have recommended special stains like the modified Gallego stain to identify the nature of mineralization in such odontogenic lesions. Krishnakumar et al. [12] reported that bone was presented as green in color, cells were pink in color, and collagen was green-pink in color which helped in the correct diagnosis facilitating the correct treatment modality. Afroze et al. [13] reported that enamel, dentine, cementum, and bone uniformly stained pink with conventional hematoxylin and eosin stains. The modified Gallego stain could be used to differentiate between these structures as enamel stained pink and dentin and bone stained green, while cementum stained red. Our case also showed eosinophilic calcifications that required special stains to differentiate their content.

The recommended treatment of odontoma includes the surgical removal of the lesion, and it is associated with a small percentage of recurrence. During surgery, especially in children, it is crucial to avoid damage to adjacent teeth and anatomic structures of the jaws [15]. Our case was also surgically removed under local anesthesia, and the patient remained disease-free on follow-up.

Hii et al. [16] have studied the expression of homeobox genes by next-generation sequencing, microarray analysis, reverse transcription-polymerase chain reaction (RT-PCR), Western blotting, in situ hybridization, and immunohistochemistry. They reported that LHX8 and DLX3 homeobox genes are expressed in odontoma. They concluded that evidence is insufficient to support any definite role of the homeobox gene in odontogenic lesions. Future research is needed to decipher the pathogenesis of such neoplasms.

## Conclusions

We have presented a unique case of an eight-year-old boy who presented with swelling in the lower back tooth region for the past few months. Clinical examination revealed a carious tooth, and a radiolucency around the impacted tooth was noted in the orthopantomogram. With careful histopathological interpretation and the use of special stains, we could conclude that it was a developing odontoma. 
